# A Step Forward Understanding Directional Limitations in Markerless Smartphone-Based Gait Analysis: A Pilot Study

**DOI:** 10.3390/s24103091

**Published:** 2024-05-13

**Authors:** Pavol Martiš, Zuzana Košutzká, Andreas Kranzl

**Affiliations:** 1 2nd Department of Neurology, Faculty of Medicine, Comenius University, 833 05 Bratislava, Slovakia; kosutzka1@uniba.sk; 2 Laboratory for Gait and Movement Analysis, Orthopedic Hospital Speising, 1130 Vienna, Austria

**Keywords:** OpenCap 2, markerless gait analysis, directional bias in motion analysis, gait kinematics, smartphone-based motion capture, timed up and go (TUG) test

## Abstract

The progress in markerless technologies is providing clinicians with tools to shorten the time of assessment rapidly, but raises questions about the potential trade-off in accuracy compared to traditional marker-based systems. This study evaluated the OpenCap system against a traditional marker-based system—Vicon. Our focus was on its performance in capturing walking both toward and away from two iPhone cameras in the same setting, which allowed capturing the Timed Up and Go (TUG) test. The performance of the OpenCap system was compared to that of a standard marker-based system by comparing spatial-temporal and kinematic parameters in 10 participants. The study focused on identifying potential discrepancies in accuracy and comparing results using correlation analysis. Case examples further explored our results. The OpenCap system demonstrated good accuracy in spatial-temporal parameters but faced challenges in accurately capturing kinematic parameters, especially in the walking direction facing away from the cameras. Notably, the two walking directions observed significant differences in pelvic obliquity, hip abduction, and ankle flexion. Our findings suggest areas for improvement in markerless technologies, highlighting their potential in clinical settings.

## 1. Introduction

Walking is part of multiple assessment tests, some of which incorporate different types of movements. One such clinical test is the Timed Up and Go test (TUG), which assesses mobility and balance. Walking is a crucial part of this assessment test. The test consists of two walking parts: the first away from the chair and the second one toward the chair. This test was initially developed by Podsiadlo and Richardson in 1991 [1] in the original version of the test named the “Get-up and Go”, which aimed to evaluate dynamic balance in elderly people [2]. This test was further successfully tested on patients with multiple disorders [3,4,5]. The TUG test highly correlates with age, socioeconomic status, and multiple comorbidities [6]. To obtain even more information from the TUG test, recent developments in movement analysis like wearables and markerless motion capture systems offer a fast way to gather detailed movement data [7,8]. These new technologies could improve how we conduct and interpret tests like the TUG without slowing down the assessment process as well as potentially enhancing our understanding and measurement of such tests without compromising evaluation time [9]. The TUG test can be further processed into individual sub-phases such as standing up from the chair, walking, turning around, walking back, and sitting down. In these sub-phases, parameters such as the speed and the quality of movement have been previously evaluated [10,11,12,13,14,15,16,17]. When using measurement tools such as cameras, the position of the cameras relative to the participant should be considered.

Markerless motion capture systems use standard video to record movement without markers. Its progress is based on recent advancements in deep learning techniques that identify body segment positions and orientation [18], potentially overcoming the accuracy limitations posed by skin artifacts associated with marker-based methods [19]. Although the latter remains the benchmark for precision, its high costs and the necessity for elaborate laboratory setups are significant drawbacks [20]. Markerless technologies and IMU sensors promise more accessible and rapid gait assessments at reduced costs. Multiple markerless systems have been tested. Ease of use has driven more simple systems ranging from one camera [21,22] to more accurate multiple-camera setups [23]. The accuracy of the markerless systems is at times plane-dependent, which could be explained by the differences in the methods and placement of the cameras [20].

OpenCap [24] is a recent addition to the field of markerless motion capture technologies. Unique for requiring only two smartphones to operate, although only iPhones are currently supported, OpenCap simplifies capturing human movement, making it broadly applicable in various settings. While it is a relatively new technology and not necessarily a major advancement, according to Uhlrich et al. [24], OpenCap can allow for assessments that may be fast enough to enable movement screens to become part of routine clinical care, allowing clinicians to track function over time as well as following an injury or surgery to benchmark rehabilitation status against preinjury measures. The first developer-led study conducted by Uhlrich et al. [24] analyzed a setup with two iPhones compared to the current golden standard (marker-based 3-dimensional motion capture system) for different tasks (walking, squatting, sit-to-stand, and drop jumps) and found a mean absolute error of 4.5° in the rotational kinematics of the lower limb across these tests. Walking toward the cameras was between 2.3 and 6.6°. They stated that the OpenCap system had similar RMSE values (2.0–10.2°) to the inertial measurement unit-based approaches and video-based systems with eight cameras. Horsak et.al. [25] confirmed the RMSE values found by Uhlrich et al. [24]. The inter-trial variability in walking analyzed by Horsak et al. [26] showed similar results to other multiple-camera markerless systems. In general, they found an increased inter-trial variability in the markerless system. OpenCap measures 3D kinematics, providing a theoretical framework for screening tests like the TUG test, which consists of movements in all three dimensions. Previous studies have already evaluated the sit-to-stand test [24] and walking toward the cameras [25]. However, as far as we know, no study has compared the accuracy of walking within the TUG test setup and the consistency in recording walking in the opposite direction to that of iPhone cameras yet.

Our study aimed to comprehensively compare the system’s precision in capturing gait from various orientations. The first goal of our study was to compare the accuracy of the OpenCap system to the golden standard marker-based system in the settings of the TUG test for walking. For this purpose, the cameras needed to be placed in a position to capture the whole area during the TUG test. The study compared the kinematic and spatial-temporal parameters measured by OpenCap and the marker-based system, utilizing correlation analysis in order to find specific parameters that are possible to estimate using the OpenCap system. The second goal was to compare the consistency of measuring gait when walking toward (WTC—walk toward cameras) and away from the cameras (WAC—walking away from cameras).

This examination is vital to thoroughly assess the OpenCap system’s full potential, focusing on its application in rapid evaluation tests such as the TUG test, shedding light on how much its ease of use and accessibility correspond with its accuracy and dependability in practical clinical settings.

## 2. Materials and Methods

### 2.1. Participants

Our study involved 10 participants including six men. One of the subjects had an asymmetrical gait pattern. The average age of the participants was 29.7 ± 8.6 (minimum: 21, maximum: 51) years, mass: 74 ± 13 kg (minimum: 49 kg, maximum: 90 kg), height 176.6 ± 11.5 cm (minimum: 160 cm, maximum: 190 cm), BMI: 23.5 ± 2 (minimum: 18.9, maximum: 25.4). All participants gave their written consent to participate in the study. The study was conducted in accordance with the Declaration of Helsinki, and the protocol was approved within a larger project by the Ethics Committee of University Hospital Bratislava (Approval number: 07/2020).

### 2.2. Measurement Setup

**Figure 1:**
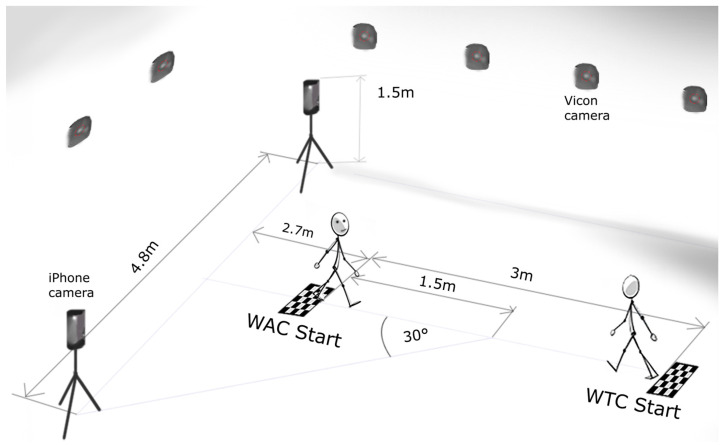
The schema of the motion capture setup. A markerless system used two iPhone cameras placed at a distance of 4.8 m from each other at an angle of 30 degrees to the center of the walking area, which was 3 m long. The marker-based setup employed 17 Vicon cameras surrounding the central walkway. One side marks the beginning of the walk toward the iPhone cameras (WTC Start), while the opposite side signifies the start of walking away from them (WAC Start).

Our experimental setup took place in the gait laboratory at the Orthopedics Hospital Speising in Vienna. We separately recorded walking movements toward (WTC) and away from the iPhone cameras (WAC). Our methodology involved a dual-system approach, utilizing marker-based and markerless camera systems (Figure 1). Five to seven recordings were conducted for each participant in each walking direction. The markerless system was positioned in accordance with the standard protocol of the 3-m TUG test, although the test itself was not recorded. 

#### 2.2.1. Markerless System

For the markerless system, we set up the OpenCap system (Neuromuscular Biomechanics Laboratory, Stanford, CA, USA, https://www.opencap.ai/) according to the recommended guidelines [27] with two iPhone cameras: an iPhone 12 and an iPhone 14. These were positioned on a tripod at the height of 1.5 m, angled downward by 5°. The iPhones were arranged to ensure that the center of the TUG area was squarely in the middle of the capture zones of both cameras. Additionally, the cameras were angled at 30° toward the center of the walking area (Figure 1). The starting position for the WTC was initiated from a stationary stance at a distance of around 5.7 m from the camera. Conversely, in WAC, the initiation point was set at 2.7 m from the camera. The recording stopped when the subject exited the recording area of the OpenCap system. OpenCap recorded the videos with default settings utilizing the OpenPose estimation algorithm, with a resolution of 720 × 1280 pixels and a frame rate of 60 Hz. OpenCap’s embedded musculoskeletal model was from Lai et al. [28] and Rajagopal et al. [29], with modified hip abductor muscle paths, according to Uhlrich et al. [24]. The OpenCap version used was from November to December 2023. 

#### 2.2.2. Marker-Based System

An optoelectronic motion capture system comprising 17 cameras (VICON, Oxford, UK) was employed for the marker-based measurement. Modified marker sets, specifically the Cleveland Clinical Marker Set (for the lower extremity) and the PlugIn Gait Model (upper extremity) [30], incorporating a total of 49 markers, were utilized. The recording frequency was set at 150 Hz. Within the Nexus software (Version 2.15, Vicon, Oxford, UK), markers underwent reconstruction, default filtering (Woltring filter, mean squared error [MSE], smoothing at 15 units) [31], and subsequent storage. Notably, a seasoned user manually designated the events of initial contact (IC) and toe-off. The marker trajectories were then used to run OpenSim’s (NIH National Center for Simulation in Rehabilitation Research, Stanford, CA, USA, https://opensim.stanford.edu) [32] inverse-kinematic tool with a musculoskeletal model from Rajagopal et al. [29]. 

### 2.3. Calculation of Parameters

**Figure A1:**
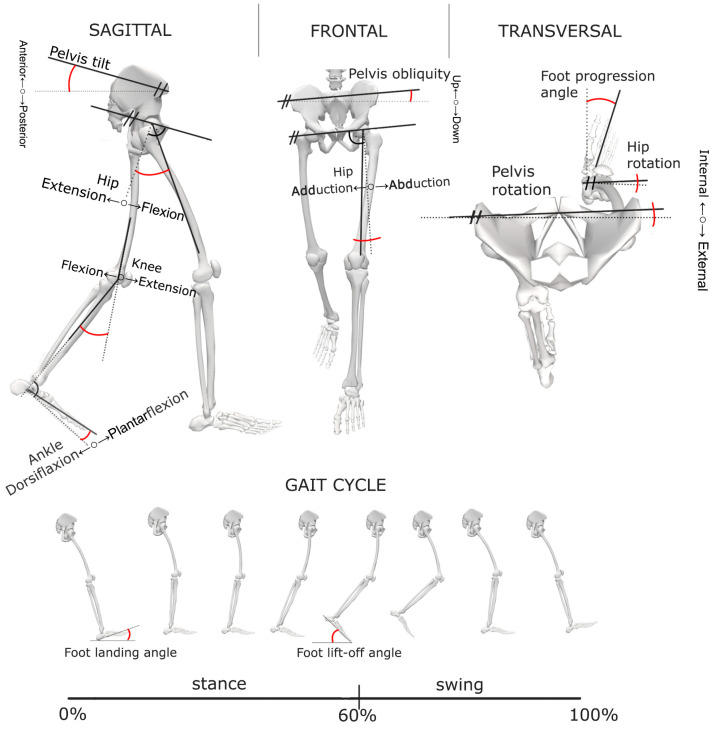
Visual representation of the kinematic parameters and gait cycle. The gait cycle starts with the initial contact of the foot at 0% of the gait cycle and ends with the following initial contact of the same foot at 100% of the gait cycle. Toe-off, which marks the transition from stance to swing phase, occurs on average at 60% of the gait cycle. The ‘side’ of the gait cycle refers to the referenced leg.

Kinematics were processed within OpenSim with the use of the same musculoskeletal model [29] for the pelvis, hip, knee, and ankle joints. The visual representation of the angles can be seen in Figure A1. In the gait cycle, ‘side’ refers to left or right strides, corresponding to the side of the pelvis being assessed and the associated leg’s hip, knee, and ankle. Stance and swing phase durations in percentage were calculated for each stride and for each side. The foot progression angle was calculated as the angle between the long axis of the foot with the axis in the direction of walking (transverse plane). The foot lift-off angle was calculated as the angle between the line of the heel–toe marker and the horizontal plane. The foot landing angle describes the same angle calculated at initial contact. Stride length was calculated as an Euclidian distance of the heel marker between two consecutive initial contacts. Walking speed was calculated by multiplying cadence with stride length. Step length was measured as the linear distance in the walking direction between the heel markers at initial contact, and step width as the distance perpendicular to the walking direction between the heel markers at initial contact. 

### 2.4. Data Analysis

Data processing and statistical analysis were conducted using MATLAB R2022b (MathWorks, Natick, MA, USA). The OpenCap data were processed using OpenCap’s web application [24]. First, we adjusted the signal from the markerless data to match the marker-based system’s frequency by interpolating the data to 150 Hz using a cubic spline function and applying a Butterworth low-pass filter of 10 Hz. Data synchronization was achieved by aligning the peak flexion of the right knee, followed by cross-correlation to adjust the entire signal. Every measurement was then individually checked and corrected if needed. After the synchronization, we cut the signal from either system to obtain the same gait cycles from both systems. From each walking recording, we eliminated the first step. We normalized all joint kinematic variables of both systems to match the 100% gait cycle. 

We employed the root mean square error (RMSE) and statistical parametric mapping (spm1d [33]) analysis for each joint kinematic variable, walking direction, and utilized system. We then extracted the kinematic and spatial-temporal parameters, averaged across the participants for the left and right sides. We utilized the Spearman correlation test in spatial-temporal and kinematic parameters across the two systems to assert the correlation. Power analysis was performed with the GPower software (Version: 3.1.9.7) with *n* = 10, α = 0.05 [34].

## 3. Results 

### 3.1. Comparing the Markerless against the Marker-Based System

**Figure 2:**
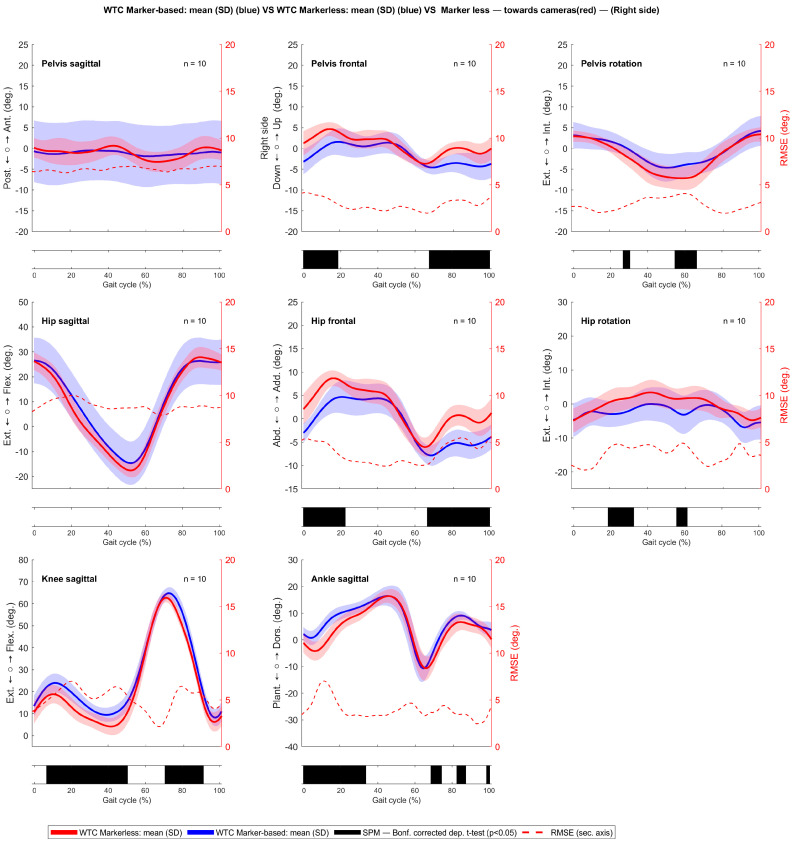
Kinematic analysis comparing the WTC for the right gait cycle using markerless (red line) and marker-based (blue line) systems, with the root mean square error (RMSE) shown as a red dotted line, and statistically significant differences, indicated by black bars beneath the respective kinematic signals, were determined by statistical parametric mapping (SPM1d) analysis at a significance level of *α* = 0.05.

The kinematic plot with SPM analysis and RMSE conducted between the markerless and marker-based systems in the WTC revealed notable distinctions in the gait cycle’s dynamics, which are visible in Figure 2. For the right gait cycle, we observed disparities in different phases of the gait cycle in the pelvic obliquity, hip abduction, hip rotation, knee flexion, and ankle flexion. On the left side in Figure A5, a similar trend could be seen. 

Additionally, in Table 1, when performing Spearman correlation of the calculated kinematic variables between the marker-based system and markerless system for WTC, we only found significant correlations for both sides in only two out of twenty-three kinematic extracted parameters: hip flexion range of motion and hip rotation at initial contact. Some kinematic parameters showed significant correlation only on one side: mean hip rotation during the stance phase, knee flexion range of motion, maximal plantar flexion, and angle lift-off. When evaluating the spatial-temporal parameters, the Spearman correlation in Table 1 showed a significant association for both legs, namely stride length (r = 1; *p* < 0.001), step length (r = 0.98; *p* < 0.001), and gait speed (r = 1; *p* < 0.001). We also observed a significant correlation with step width (0.94; *p* < 0.001). Stance phase duration in the WTC for the right side was 60.2 ± 2.3% and 60.3 ± 2.0% for the left side.

Similar, although slightly worse, results could be observed for the WAC in Figure A6 and Figure A7, where the comparison revealed a different propagation of kinematics in the pelvic list, hip abduction, knee flexion, and ankle flexion. Compared to the WTC, the differences in the WAC seemed to be more exaggerated, which was confirmed in Table A3, where the difference in hip obliquity ROM > 9° (<2° in WTC), hip abduction ROM > 7° (<3° in WTC), knee flexion ROM > 6° (<3° in WTC), and knee flexion at IC > 6° (3° in WTC). We did not see a significant correlation for any kinematic parameter. Spatial-temporal parameters showed significant correlations similar to the WTC. Stance phase duration in the WAC was 60.4 ± 1.8% for the right side and 60.7 ± 2.0% for the left side.

### 3.2. Case Section

**Figure 3:**
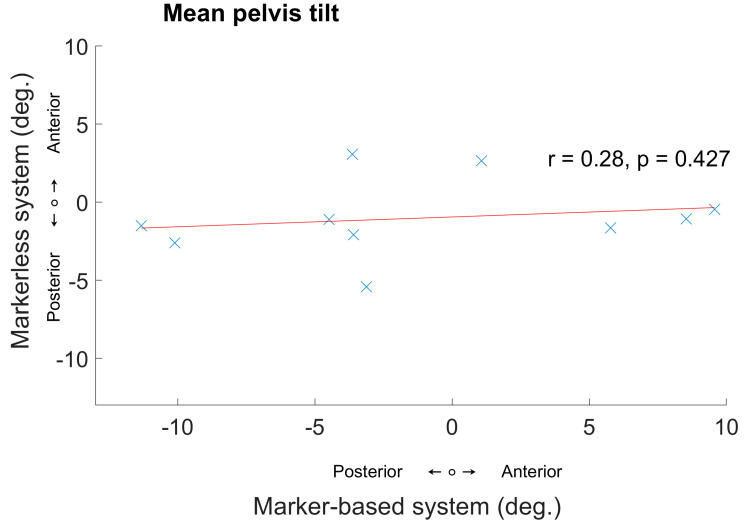
Mean pelvic tilt of each participant. A comparison between the marker-based and the OpenCap system for each participant.

In our comparative analysis of pelvis tracking data between marker-based and markerless systems, notable differences emerged in the standard deviations (SD) of the measurements. Specifically, the captured mean pelvic tilt in Table 1 displayed a larger standard deviation (7.2°) in the marker-based system, signifying a wider variability in pelvic movement. In contrast, data obtained from the markerless system demonstrated a smaller standard deviation in the mean pelvic tilt (2.4°), indicating more consistent pelvis movement data with less variability. While we saw a wider range of pelvis tilt captured by the marker-based system (Figure 3), we did not observe the expected correlated values in the markerless system (the same was true for the left side). We explored these findings through two case presentations.

#### 3.2.1. Visible Lordosis

**Figure 4:**
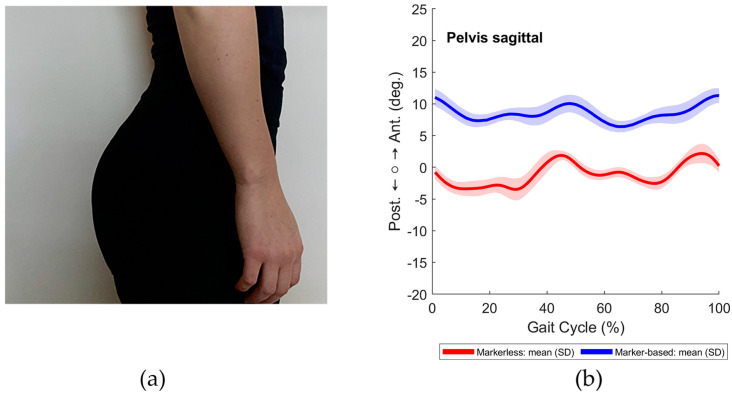
(**a**) Sagittal view of the pelvis. (**b**) The movement of pelvic tilt during the right gait cycle: marker-based (blue line) compared to markerless (red line).

The first case illustrated in Figure 4 presents a participant with a visible anterior pelvic tilt. The marker-based analysis, represented by the blue line in Figure 4, indicated a pelvic tilt of around 10 degrees visible over the whole gait cycle. Despite the apparent similarity in movement patterns, the markerless system failed to accurately capture the anterior pelvic tilt, recording a value approximating 0°.

#### 3.2.2. Pelvic Movement in One Participant with Asymmetric Gait Pattern

**Figure 5:**
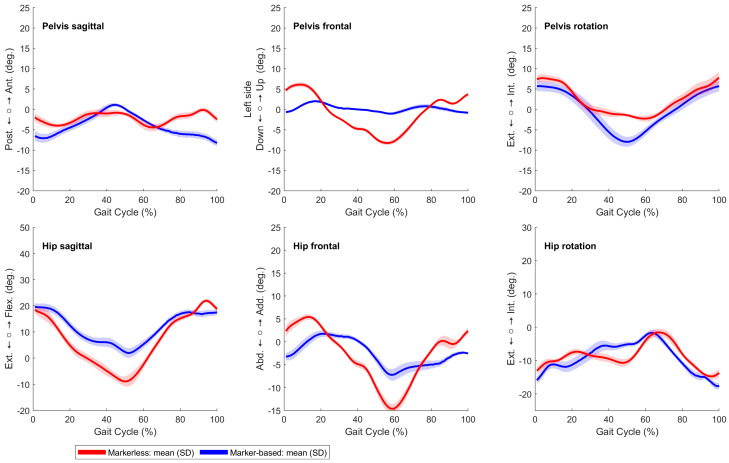
Pelvic and hip movement (left side) of marker-based (blue line) and markerless (red line) systems.

The second case presents a participant with an asymmetric gait pattern. He has reduced hip extension and compensatory movement in the pelvis. Looking at the pelvic movement from the markerless system in Figure 5 for the left gait cycles, there was less anterior tilt of the pelvis at the end of the single support phase than in the mgiarker-based data. The marker-based system showed less hip extension in the terminal stance phase, while the markerless system presented a normal hip extension value. The ROM in the frontal plane in the pelvis and hip was greater with the markerless system, whereas the ROM was smaller in the transverse plane. From a clinical point of view, we would expect a reduced extension in the terminal stance phase for this participant, as seen by the marker-based system.

### 3.3. Comparing Walking Directions in the Markerless System

The comparison of the averaged RMSE for the left and right legs using the markerless and marker-based systems revealed significant differences in kinematic analysis (Table 2). The markerless system showed a grand mean RMSE of 4.5 ± 2.9°, while the marker-based system had a notably lower mean of 1.0 ± 0.8°. Differences between the two systems were particularly notable in pelvic obliquity (5.8 ± 2.0° vs. 0.3 ± 0.2°), hip flexion (4.6 ± 2.9° vs. 1.7 ± 1.4°), hip abduction (6.6 ± 3.9° vs. 0.63 ± 0.47°), hip rotation (3.6 ± 2.5° vs. 1.0 ± 0.7°), knee flexion (3.6 ± 2.5° vs. 1.3 ± 1.2°), and ankle flexion (8.5 ± 4.6° vs. 0.8 ± 0.82°). 

The analysis further detailed the RMSE for the WTC and WAC directions between the two systems, emphasizing greater discrepancies in the WAC (grand mean 4.9 ± 3.3° vs. 6.6 ± 4.6°), especially in pelvic obliquity (2.8 ± 2.1° vs. 5.3 ± 3.2°), hip abduction (vs 3.5 ± 2.5° vs. 6.1± 4.2°), and ankle flexion (4.7 ± 3.4° vs. 11.9 ± 6.4°). Table A1 demonstrates notable discrepancies in the spatial-temporal and kinematic parameters between directions in the markerless system, with most variables showing differences greater than 3°. In contrast, Table A2, detailing the marker-based system, revealed smaller variations, with differences under 2°. Additionally, the markerless system lacked significant correlation across 18 kinematic parameters for both directions.

**Figure 6:**
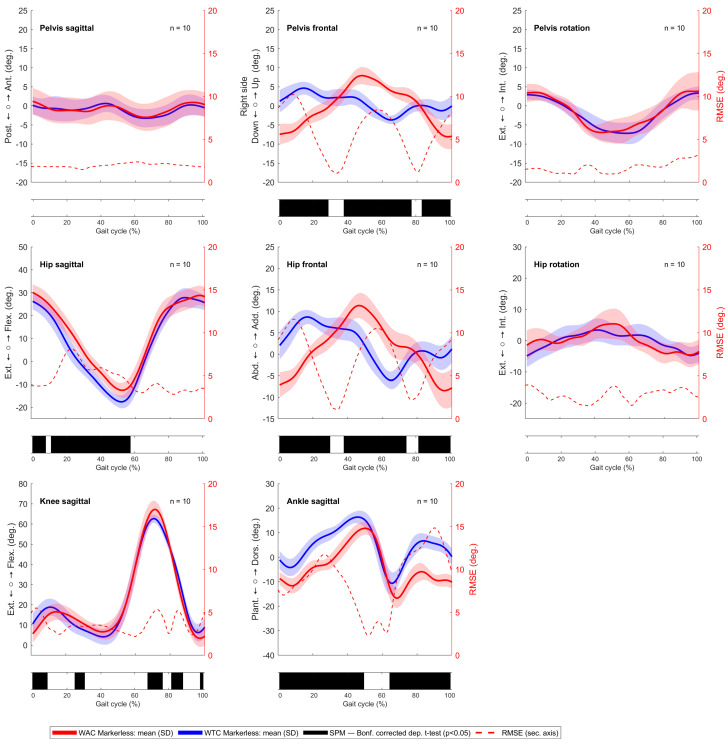
Kinematic comparison of the right gait cycle between the WTC (blue line) and WAC (red line) using the markerless system. Root mean square error (RMSE) is shown as a red dotted line, and statistically significant differences, indicated by black bars beneath the respective kinematic signals, were determined by statistical parametric mapping (SPM1d) analysis at a significance level of *α* = 0.05.

**Figure A2:**
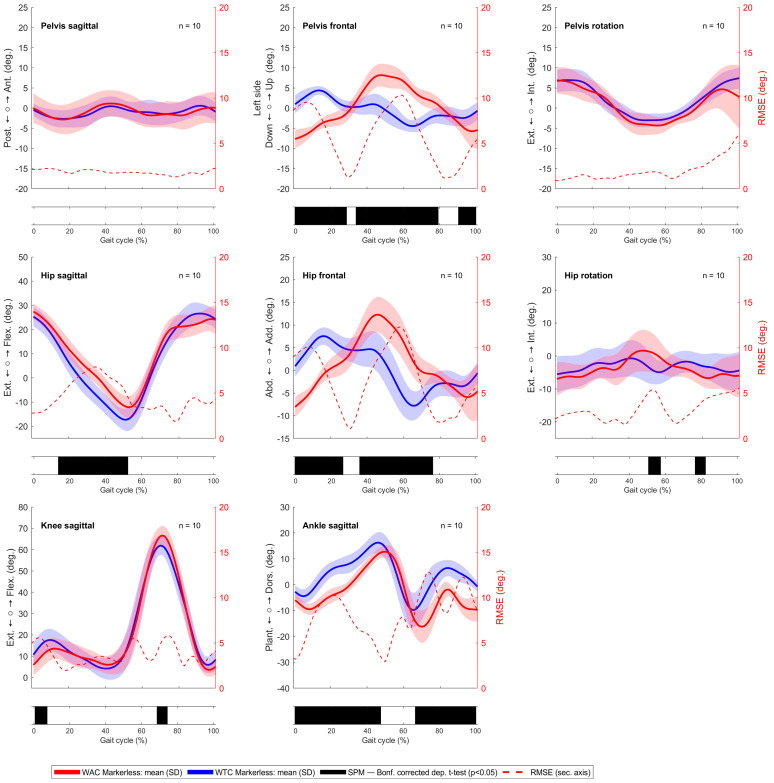
Kinematic comparison between the WTC (red) and WAC (blue) in the left leg in the markerless system. Root mean square error (RMSE) is shown as a red dotted line, and statistically significant differences, indicated by black bars beneath the respective kinematic signals, were determined by statistical parametric mapping (SPM1d) analysis at a significance level of *α* = 0.05.

**Figure A3:**
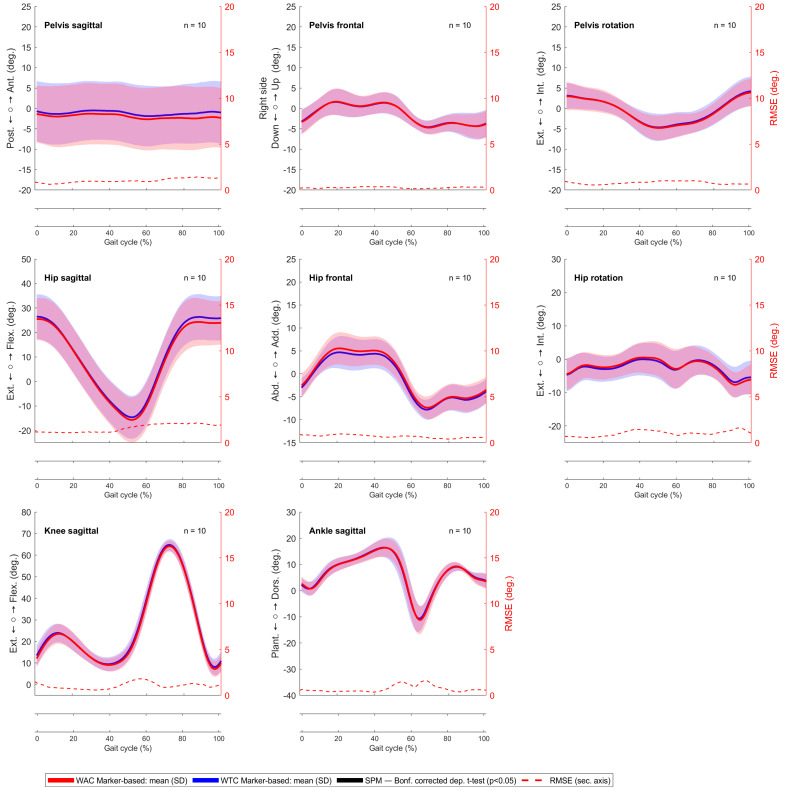
Kinematic comparison between the WTC (red) and WAC (blue) in the right leg in the marker-based system. Root mean square error (RMSE) is shown as a red dotted line, and statistically significant differences, indicated by black bars beneath the respective kinematic signals, were determined by statistical parametric mapping (SPM1d) analysis at a significance level of *α* = 0.05.

**Figure A4:**
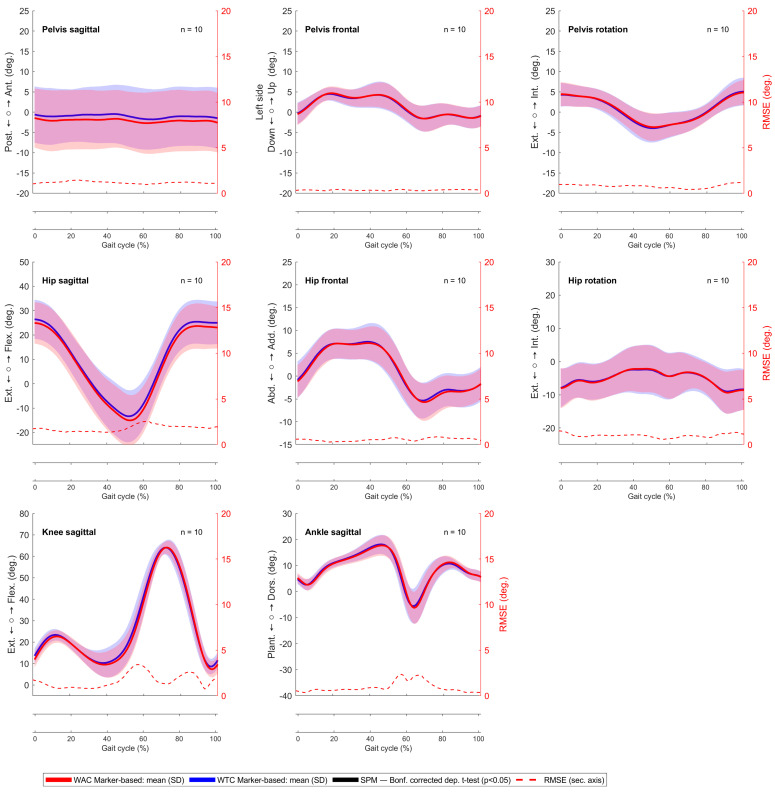
Kinematic comparison between the WTC (red) and WAC (blue) in the left leg in the marker-based system. Root mean square error (RMSE) is shown as a red dotted line, and statistically significant differences, indicated by black bars beneath the respective kinematic signals, were determined by statistical parametric mapping (SPM1d) analysis at a significance level of *α* = 0.05.

**Figure A5:**
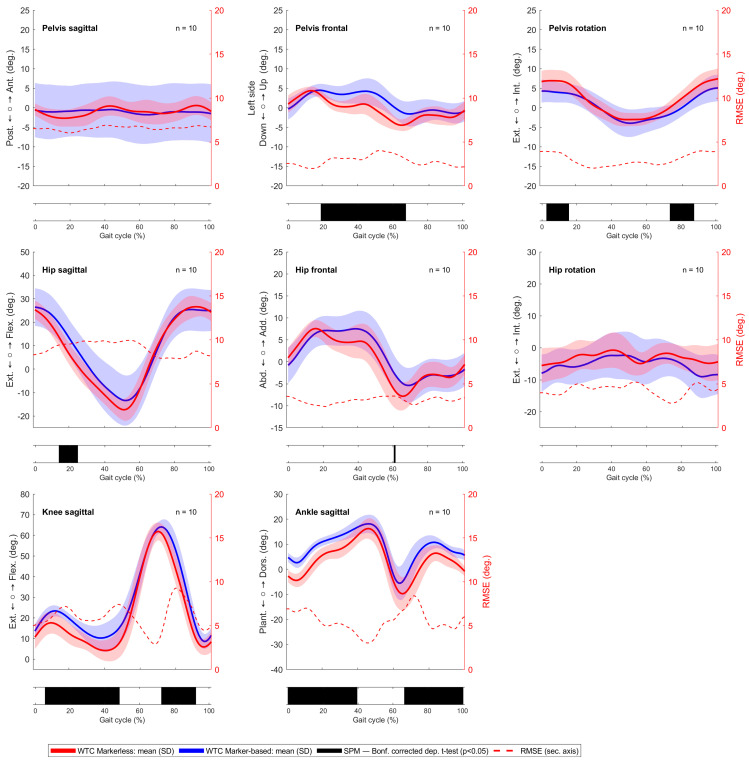
Kinematic comparison between the markerless (red line) and marker-based (blue line) systems in the WTC in the left leg, with the root mean square error (RMSE) shown as a red dotted line, and statistically significant differences, indicated by black bars beneath the respective kinematic signals, were determined by statistical parametric mapping (SPM1d) analysis at a significance level of *α* = 0.05.

**Figure A6:**
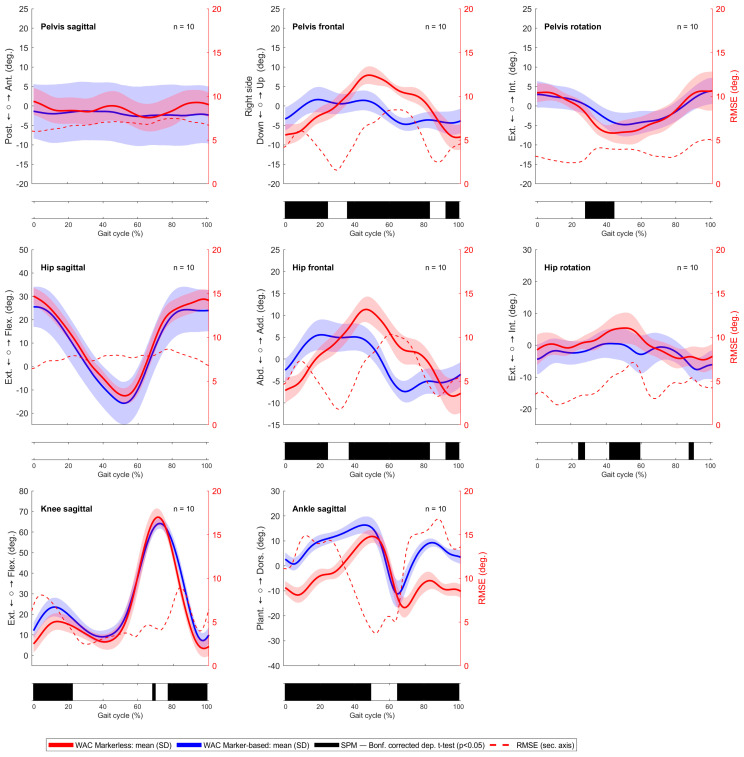
Kinematic comparison between the markerless (red line) and marker-based (blue line) systems in the WAC in the right leg, with the root mean square error (RMSE) shown as a red dotted line, and statistically significant differences, indicated by black bars beneath the respective kinematic signals, were determined by statistical parametric mapping (SPM1d) analysis at a significance level of *α* = 0.05.

**Figure A7:**
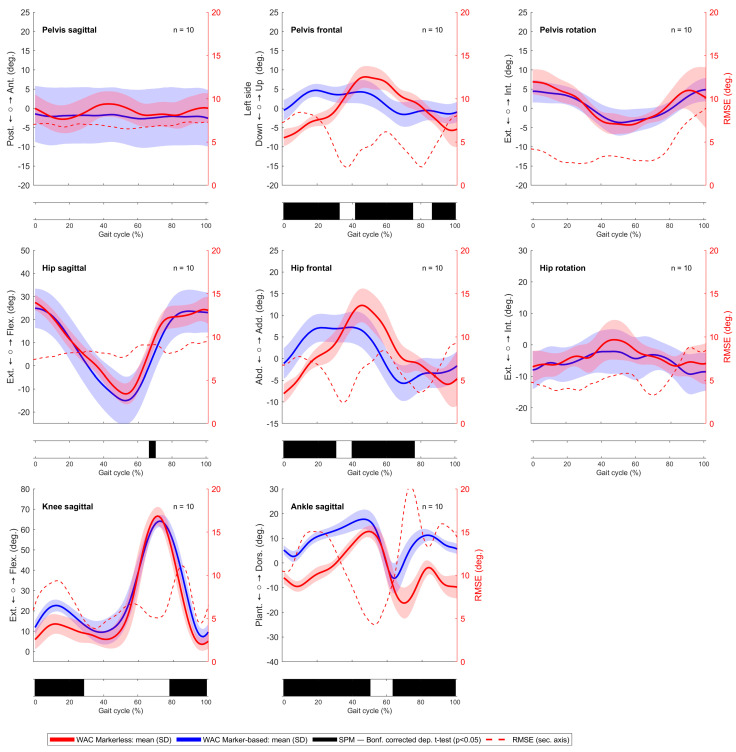
Kinematic comparison between markerless (red line) and marker-based (blue line) systems in the WAC in the left leg, with the root mean square error (RMSE) shown as a red dotted line, and statistically significant differences, indicated by black bars beneath the respective kinematic signals, were determined by statistical parametric mapping (SPM1d) analysis at a significance level of *α* = 0.05.

This difference was confirmed by the observed kinematic analysis between the WTC and WAC in the markerless system, as shown in the SPM1d analysis in Figure 6 for the right side (see Figure A2 for the left side). We did not observe such differences in the marker-based system (Figure A3 and Figure A4).

## 4. Discussion

The objective of this study was to assess and compare the outcomes of the markerless system while walking toward and away from the iPhone cameras to those of the marker-base system in the recording setup, which was able to capture the TUG test. For this reason, we utilized statistical parametric mapping and correlation analysis. 

First, we evaluated the accuracy in the WTC direction, as this is the standard protocol used in previous studies. The calculated average grand mean error (RMSE) between the OpenCap and the marker-based systems in the kinematics of the WTC was 4.87°, similar or slightly worse to the results from Horsak et al. without the subtalar joint (4.61°) [26]. Uhlrich et al. presented a lower grand mean error of 4.1° [24]. However, the sole comparison of the RMSE did not tell us how the markerless system detects variability in different kinematic motions. Horsak et al. compared different types of walking, resulting in OpenCap having a worse performance in abnormal walking types [25], and in a recent study [26], Horsak et al. compared the inter-trial variability between the OpenCap and marker-based systems, resulting in OpenCap having a 6.6–22% increase in inter-trial variability in the averaged joint kinematics compared to the marker-based system. To better understand the differences in capturing the kinematics, we calculated the frequently used kinematics and spatial-temporal parameters and then calculated their correlation to the marker-based system. We found an excellent correlation in spatial-temporal parameters, especially when measuring stride length, which was almost identical to the marker-based system (r = 1; *p* < 0.001) as well as a good correlation with the step width (r = 0.94; *p* < 0.001). When evaluating the kinematic parameters in Table 2, however, most of the parameters failed to record a significant correlation.

These observations led us to examine the differences in the individual cases further. In the first case, a detailed examination of pelvic tilt revealed the markerless system’s problem in detecting lordosis. We observed the markerless system’s tendency to report pelvic tilt measurements as close to zero degrees, indicating a bias toward a neutral pelvic position. This standardization effect persisted even in the case of significant lordosis, implying that the markerless system might not accurately represent deviations from a neutral pelvic tilt. Given the coordinate definitions used in the OpenSim calculations, while some underestimation compared to actual pelvic tilt was expected, a value around 0° suggests a considerable underrepresentation of the actual pelvic tilt. The second case revealed the markerless system’s problem in capturing kinematic motion in a participant with an asymmetric gait pattern. This points to the markerless system’s lack of sensitivity in capturing nuanced gait dynamics, a critical aspect often necessary for clinical assessments. The definition of the pelvis segment is often difficult for markerless systems. Wren et al. [35] found high RMSE values for the sagittal pelvis movement in the markerless system. In their discussion, they speculated that a different coordinate system might influence these results. However, the case presentation in our study demonstrates that the patterns were not the same for both systems used. 

The second goal of this study was to assess the possibility of the OpenCap system recording the same results when the participant was turned away from the iPhone cameras. First, we compared the differences across all participants in the different walking directions. Overall, the grand mean error between the two directions in the markerless system showed a mean difference of 4.47° across all joints. Among those, we observed a clinically significant difference (>5 degrees) in pelvic obliquity (5.75 ± 3.26°), hip abduction (6.6 ± 3.92°), and ankle flexion (8.51 ± 4.59°). To differentiate the effect of the error caused by the sensor from the possible different walking patterns between the trials, we measured the same trials in parallel with the marker-based system, which recorded a grand mean RMSE of only 0.96°, and none of the joints presented an error higher than 2°. SPM analysis in the kinematics of joints visualized these differences mainly in pelvic obliquity, hip abduction, hip flexion, knee flexion, and ankle flexion. We separately compared the markerless to a marker-based system with the same gait cycles in different walking directions. Our results confirmed our hypothesis: that recording participants facing the camera opposite will yield different results. Better accuracy was obtained in the direction with the participants facing the cameras, with lower RMSE values in the WTC, particularly in the pelvic obliquity (2.8° to 5.3°, respectively), pelvic rotation (2.9° to 3.8°, respectively), hip abduction (3.5° to 6.1°, respectively), and ankle flexion (4.7° to 11.9°, respectively). Max RMSE values were also lower in the WTC. 

The OpenCap system can use multiple cameras to take measurements, but all cameras must be positioned within an angular range of −90° to 90° in front of the calibrator during the calibration step [24]. This range is necessary because the calibration process requires every camera to have a clear view of the checkerboard calibrator. Although adding more cameras within this range is possible, experiments with configurations of up to five cameras at angles of ±70°, ±45°, and 0° have shown that this configuration does not significantly improve the measurement accuracy [24]. Additionally, incorporating more cameras increases the system’s complexity, which diminishes the advantages of its easy setup and quick measurement capabilities.

We would like to acknowledge and address certain limitations including small sample sizes, the manual detection of gait events, different filtering, and the challenges in data synchronization. As the study was designed as a pilot study with 10 participants, it is important to look at the power of the individual parameters in order to assess the robustness of the results/individual parameters. The results showed low to high power values depending on the parameter. However, the values should not be overestimated in this respect because of our small sample. There was a difference in the frequency of the measured signals from the cameras of 60 Hz to 150 Hz. For that reason, an interpolation of the data was conducted. We did not expect this to alter the results in the shape of the kinematics significantly. More differences could arise from the time synchronization of the data between the two sensors. We synchronized the data within the right leg, which may have affected the results mainly on the left side. These factors could affect the precision of the analysis. Despite these limitations, our findings revealed robust differences. Addressing these issues would likely refine our results without diminishing the clear distinctions we observed in the data. 

Overall, markerless measurement systems are still in the early phase of their development, but even at this moment, they can provide clinicians with fast, low-cost screening tools that can enrich traditional tests like the TUG test, which has previously been measured only with a stopwatch in the past. However, clinicians have to consider the capabilities of these devices. To this day, there is no consensus over how to ideally set up markerless systems. Analyzing the TUG test, in particular, requires a 3-dimensional approach because, in addition to walking straight ahead, it also includes turns in the execution of the movement. For this reason, we tested the OpenCap system in this setup. To apply OpenCap to measuring clinical tests like the TUG, we recommend being careful when merging walking directions. Pose estimation algorithms in markerless are biased regarding the position and distances from the used cameras [36]. Available open-source training datasets have never been designed with biomechanical applications in mind [18]. The two directions should not present significant differences in the kinematic parameters, as was demonstrated for the hip, knee, and ankle in healthy and hemiparesis due to stroke [10,11]; furthermore, as we have shown, the OpenCap technology presents different results in different directions, and the correlations with the marker-based system in kinematic parameters are not clear.

## 5. Conclusions

The OpenCap system demonstrates promising accuracy in capturing key spatial-temporal gait parameters including step length and step width, making it a valuable tool in fields such as rehabilitation, neurology, sports science, or biomechanical research. However, its limitations in providing a comprehensive kinematic analysis highlight areas for improvement. Particularly, when conducting detailed screening analyses that focus on kinematic parameters of the pelvis or hip joints, the variability in accuracy presented by the OpenCap system warrants careful consideration. Such variability may affect the reliability of assessments in clinical or research settings, especially for conditions or studies prioritizing these anatomical areas. Moreover, the OpenCap system at the current stage of development has exhibited inconsistencies in capturing movements directed away from the camera setup, as observed in tasks like the Timed Up and Go test. This limitation is indicative of broader challenges faced by markerless motion capture technologies. Consequently, researchers and clinicians should remain cautious about the influence of walking direction on data accuracy.

These observations suggest that while the OpenCap system offers valuable insights into human gait, its integration into clinical practices demands a clear recognition of its limitations. Addressing these challenges will be crucial for leveraging the full potential of markerless motion capture technologies in advancing healthcare and research.

## Figures and Tables

**Table 1:** Spatial-temporal and kinematic parameters for WTC between the marker-based and markerless system.

Parameter	Marker-Based		Markerless		Difference		Spearman Correlation		Power	
	Right	Left	Right	Left	Right	Left	Right	Left	Right	Left
	Mean (SD)	Mean (SD)	Mean (SD)	Mean (SD)	Mean (SD)	Mean (SD)	Corr (*p*-value)	Corr (*p*-value)	power	power
Stride length (m)	1.31 (0.12)	1.31 (0.13)	1.31 (0.12)	1.31 (0.13)	0.00 (0.01)	0.00 (0.01)	**1.00 ** (<0.001) *	**1.00 ** (<0.001) *	**0.05**	**0.05**
Step width (m)	0.09 (0.02)	0.10 (0.02)	0.10 (0.02)	0.11 (0.03)	0.01 (0.01)	0.01 (0.01)	**0.94 ** (<0.001) *	**0.95 ** (<0.001) *	**0.269**	**0.188**
Step length (m)	0.65 (0.06)	0.67 (0.07)	0.65 (0.06)	0.66 (0.07)	0.00 (0.01)	0.00 (0.00)	**0.98 ** (<0.001) *	**0.99 ** (<0.001) *	**0.05**	**0.069**
Gait speed (m/s)	1.24 (0.15)	1.25 (0.15)	1.23 (0.15)	1.24 (0.15)	0.00 (0.01)	0.00 (0.01)	**1.00 ** (<0.001) *	**0.99 ** (<0.001) *	**0.054**	**0.054**
Mean pelvis tilt (°)	−1.1 (7.2)	−1.0 (7.0)	−1.0 (2.4)	−1.0 (2.3)	0.1 (7.2)	0.0 (7.1)	0.28 (0.43)	0.20 (0.58)	0.05	0.05
Mean pelvis obliquity (°)	−1.5 (2.0)	1.4 (2.0)	0.7 (0.7)	−0.4 (0.6)	2.1 (2.0)	1.9 (1.9)	0.16 (0.66)	0.39 (0.26)	0.939	0.812
Pelvis obliquity ROM (°)	8.3 (2.8)	8.3 (2.9)	9.6 (2.5)	10.1 (2.4)	1.3 (4.7)	1.7 (4.7)	−0.42 (0.23)	−0.43 (0.22)	0.282	0.473
Pelvis obliquity at IC (°)	−3.1 (3.0)	−0.2 (2.7)	1.4 (2.9)	1.1 (2.2)	4.4 (3.4)	1.3 (2.7)	0.15 (0.68)	0.31 (0.39)	0.989	0.315
Pelvis rotation ROM (°)	11.3 (3.9)	11.4 (3.7)	11.8 (2.7)	11.7 (2.8)	0.5 (4.3)	0.3 (4.4)	0.18 (0.63)	0.32 (0.37)	0.069	0.057
Mean pelvis Rotation (°)	−0.5 (1.8)	0.6 (2.0)	−1.9 (1.7)	2.0 (1.7)	1.3 (2.0)	1.4 (2.0)	0.24 (0.51)	0.22 (0.54)	0.615	0.561
Hip flexion ROM (°)	43.4 (4.6)	41.2 (8.8)	46.9 (3.6)	45.8 (6.7)	3.5 (2.9)	4.6 (4.9)	**0.64 ** (0.05) *	**0.65 ** (0.05) *	**0.653**	**0.372**
Hip flexion at IC (°)	26.5 (9.2)	26.4 (8.0)	26.0 (3.5)	25.1 (3.9)	0.4 (8.9)	1.3 (9.4)	0.22 (0.54)	−0.21 (0.56)	0.053	0.083
Maximal hip Extension (°)	−15.0 (8.8)	−13.9 (10.6)	−17.9 (2.8)	−18.0 (4.8)	2.9 (9.4)	4.1 (10.8)	−0.16 (0.66)	0.03 (0.95)	0.184	0.243
Hip abduction ROM (°)	13.7 (3.0)	14.6 ( 4.0)	15.7 (3.0)	16.5 (3.5)	2.1 (4.2)	1.9 (5.2)	0.02 (0.97)	−0.04 (0.92)	0.469	0.296
Hip abduction at IC (°)	−2.8 (2.0)	−0.6 (4.0)	2.3 (3.2)	1.2 (2.2)	5.1 (3.6)	1.8 (3.8)	−0.12 (0.76)	0.25 (0.49)	0.999	0.311
Hip rotation ROM (°)	11.5 (2.8)	11.0 (3.4)	11.7 (2.3)	11.4 (2.3)	0.2 (3.2)	0.4 (3.2)	0.27 (0.45)	0.37 (0.30)	0.056	0.067
Hip rotation at IC (°)	−4.6 (4.9)	−7.8 (5.8)	−4.8 (3.5)	−5.5 (5.4)	0.2 (2.7)	2.3 (4.3)	**0.87 ** (0.00) *	**0.75 ** (0.02) *	**0.052**	**0.213**
Mean hip rotation—stand phase (°)	−2.0 (4.0)	−4.4 (6.0)	0.7 (3.1)	−3.2 (4.2)	2.7 (2.8)	1.3 (4.7)	0.65 (0.05) *	0.58 (0.09)	0.695	0.106
Knee flexion ROM (°)	58.5 (3.2)	58.5 (4.0)	60.3 (4.4)	61.2 (4.2)	1.8 (2.8)	2.7 (3.7)	0.55 (0.10)	0.73 (0.02) *	0.268	0.459
Knee flexion at IC (°)	14.2 (4.8)	14.3 (3.4)	11.2 (5.1)	11.3 (5.7)	3.0 (3.3)	3.0 (5.3)	0.79 (0.01) *	0.37 (0.30)	0.401	0.400
Maximal knee extension —stand phase (°)	8.7 (2.9)	8.6 (3.9)	3.8 (3.6)	2.5 (2.4)	4.9 (3.3)	6.1 (4.2)	0.56 (0.10)	−0.09 (0.81)	0.993	0.949
Maximal knee flexion (°)	65.2 (2.6)	65.3 (3.4)	63.4 (2.0)	63.4 (3.9)	1.8 (2.0)	1.9 (4.3)	0.33 (0.35)	0.19 (0.61)	0.577	0.310
Ankle flexion ROM (°)	28.7 (4.0)	25.9 (4.4)	29.3 (3.0)	28.9 (5.2)	0.6 (3.9)	3.1 (4.7)	0.37 (0.30)	0.39 (0.26)	0.076	0.416
Ankle flexion at IC (°)	1.9 (2.6)	4.4 (1.8)	−1.5 (3.7)	−3.0 (2.5)	3.4 (3.3)	7.5 (3.0)	0.43 (0.22)	−0.10 (0.79)	0.828	1.000
Maximum stance dorsiflexion (°)	16.9 (3.4)	18.4 (3.5)	17.0 (2.3)	17.0 (3.7)	0.2 (3.7)	1.4 (3.2)	0.15 (0.58)	0.44 (0.20)	0.051	0.220
Maximum swing dorsiflexion (°)	9.4 (1.7)	11.3 (2.7)	8.1 (2.8)	7.7 (3.3)	1.4 (3.0)	3.6 (4.2)	0.33 (0.35)	−0.07 (0.86)	0.267	0.634
Maximum plantar flexion (°)	−11.8 (5.1)	−7.4 (6.0)	−12.3 (4.0)	−11.9 (6.3)	0.5 (3.6)	4.5 (3.9)	0.62 (0.06)	0.77 (0.01) *	0.061	0.541
Foot progression angle (°)	10.2 (3.1)	10.0 (5.3)	5.5 (3.4)	6.1 (6.3)	4.8 (4.1)	3.8 (5.6)	0.26 (0.47)	0.16 (0.66)	0.941	0.705
Foot lift-off angle (°)	61.2 (5.3)	60.7 (5.3)	59.3 (7.8)	59.5 (6.2)	1.9 (7.0)	1.2 (5.1)	0.12 (0.76)	0.73 (0.02) *	0.122	0.090
Foot landing angle (°)	11.9 (3.8)	12.4 (2.3)	6.0 (3.4)	4.8 (3.1)	5.9 (3.6)	7.6 (3.4)	0.42 (0.23)	−0.02 (0.97)	0.995	1.000

ROM—range of motion, IC—initial contact, * *p*-value < 0.05, bold letters indicate *p*-value < 0.05 on both sides.

**Table 2:** The RMSE between the WTC and WAC in both systems with an RMSE comparison of the markerless and marker-based systems for both the WTC and WAC averaged across both sides.

	Pelvic Tilt	Pelvic Obliquity	Pelvic Rotation	Hip Flexion	Hip Adduction	Hip Rotation	Knee Flexion	Ankle Flexion	
	Mean (SD)	Mean (SD)	Mean (SD)	Mean (SD)	Mean (SD)	Mean (SD)	Mean (SD)	Mean (SD)	
MEAN RMSE (WTC/WAC)									GRAND MEAN
Markerless (°)	1.9 (1.3)	5.8 (3.2)	1.9 (2.0)	4.6 (2.9)	6.6 (3.9)	3.0 (2.4)	3.6 (2.5)	8.5 (4.6)	4.5 (2.9)
Marker-based (°)	1.1 (0.9)	0.31 (0.2)	0.8 (0.6)	1.7 (1.4)	0.6 (0.5)	1.0 (0.7)	1.3 (1.2)	0.8 (0.8)	1.0 (0.8)
Difference (°)	0.8 (0.4)	5.4 (3.0)	1.1 (1.4)	2.9 (1.5)	6.0 (3.4)	2.0 (1.6)	2.3 (1.3)	7.7 (3.8)	3.5 (2.1)
MEAN RMSE (markerless/ marker-based)									
WAC (°)	6.9 (3.6)	5.3 (3.2)	3.8 (5.3)	8.0 (5.1)	6.1 (4.2)	4.7 (4.8)	5.8 (4.5)	11.9 (6.4)	6.6 (4.6)
WTC (°)	6.6 (3.6)	2.8 (2.1)	2.9 (2.0)	8.9 (5.6)	3.5 (2.5)	4.0 (3.0)	5.6 (4.2)	4.7 (3.4)	4.9 (3.3)
Difference (°)	0.3 (0.0)	2.5 (1.1)	0.9 (3.3)	−0.9 (−0.5)	2.6 (1.7)	0.7 (1.8)	0.2 (0.3)	7.2 (3.0)	1.7 (1.3)
MAX RMSE (markerless/ marker-based)									
WAC (°)	10.9 (3.4)	10.8 (2.3)	10.1 (12.0)	14.7 (5.5)	13.1 (5.2)	12.2 (8.0)	13.3 (5.2)	22.9 (7.2)	13.5 (6.1)
WTC (°)	9.6 (3.4)	6.0 (2.2)	6.1 (1.8)	14.2 (5.2)	7.6 (2.4)	8.5 (2.8)	10.8 (5.2)	10.9 (4.0)	9.2 (3.4)
Difference (°)	1.3 (0.0)	4.8 (0.1)	4.0 (11.2)	0.5 (0.3)	5.5 (2.8)	3.7 (5.2)	2.8 (0.0)	12.0 (3.2)	4.3 (2.7)

## Data Availability

Data are contained within the article.

## Appendix A

**Table A1:** Comparison of the kinematic parameters comparison between the WTC and WAC in the markerless system.

Parameter	WTC		WAC		Difference		Spearman Correlation		Power	
	Right	Left	Right	Left	Right	Left	Right	Left	Right	Left
	Mean (SD)	Mean (SD)	Mean (SD)	Mean (SD)	Mean (SD)	Mean (SD)	Corr (*p*-value)	Corr (*p*-value)		
Stride length (m)	1.30 (0.12)	1.30 (0.12)	1.30 (0.12)	1.28 (0.12)	0.01 (0.02)	0.02 (0.04)	**0.98 ** **(<0.001) ***	**0.89 ** **(0.001) ***	**0.050**	**0.067**
Step width (m)	0.09 (0.02)	0.09 (0.02)	0.12 (0.02)	0.11 (0.02)	0.02 (0.01)	0.02 (0.01)	**0.83 ** **(0.01) ***	**0.85 ** **(0.004) ***	**0.987**	**0.803**
Step length (m)	0.65 (0.06)	0.65 (0.06)	0.63 (0.07)	0.64 (0.06)	0.02 (0.05)	0.00 (0.01)	**0.68 ** **(0.04) ***	**0.96 ** **(<0.001) ***	**0.139**	**0.072**
Gait speed (m/s)	1.23 (0.13)	1.24 (0.14)	1.21 (0.13)	1.23 (0.14)	0.02 (0.04)	0.01 (0.02)	**0.92 ** **(<0.001) ***	**0.99 ** **(<0.001) ***	**0.072**	**0.055**
Mean pelvis tilt (°)	−1.0 (2.4)	−1.0 (2.3)	−0.8 (3.4)	−0.9 (3.3)	0.2 (2.2)	0.1 (2.0)	0.61 (0.07)	0.66 (0.04) *	0.054	0.054
Mean pelvis obliquity (°)	0.7 (0.7)	−0.4 (0.6)	−0.0 (0.9)	0.3 (0.6)	0.7 (0.8)	0.7 (0.6)	0.43 (0.22)	0.55 (0.10)	0.673	0.906
Pelvis obliquity ROM (°)	9.6 (2.5)	10.1 (2.4)	17.4 (4.1)	18.3 (3.6)	7.8 (4.9)	8.2 (3.7)	0.14 (0.71)	0.38 (0.28)	0.999	0.999
Pelvis obliquity at IC (°)	1.4 (2.9)	1.1 (2.2)	−7.4 (2.7)	−7.6 (2.2)	8.8 (1.8)	8.7 (1.6)	0.44 (0.20)	0.68 (0.04) *	1.000	1.000
Pelvis rotation ROM (°)	11.8 (2.7)	11.7 (2.8)	14.4 (3.5)	17.4 (4.6)	2.6 (3.2)	5.6 (5.1)	0.47 (0.18)	0.14 (0.71)	0.636	0.978
Mean pelvis rotation (°)	−1.9 (1.7)	2.0 (1.7)	−1.5 (2.1)	0.9 (1.8)	0.4 (1.3)	1.1 (1.0)	**0.77 ** **(0.01) ***	**0.88 ** **(0.002) ***	**0.090**	**0.425**
Hip flexion ROM (°)	46.9 (3.6)	45.8 (6.7)	43.9 (3.4)	41.5 (5.7)	3.0 (2.5)	4.4 (3.3)	**0.79 ** **(0.01) ***	**0.84 ** **(0.004) ***	**0.674**	**0.492**
Hip flexion at IC (°)	26.0 (3.5)	25.1 (3.9)	29.9 (3.6)	27.2 (3.3)	3.9 (1.7)	2.1 (3.7)	0.92 (<0.001) *	0.42 (0.23)	0.870	0.372
Hip sagittal—max extension (°)	−17.9 (2.8)	−18.0 (4.8)	−13.0 (3.0)	−13.0 (3.9)	4.9 (2.8)	5.0 (1.5)	0.52 (0.133)	0.92 (<0.001) *	0.997	0.888
Hip abduction ROM (°)	15.7 (3.0)	16.5 (3.5)	21.2 (5.5)	23.7 (4.2)	5.5 (6.9)	7.2 (4.7)	−0.02 (0.97)	0.25 (0.49)	0.899	0.999
Hip abduction at IC (°)	2.3 (3.2)	1.2 (2.2)	−7.0 (2.8)	−7.9 (2.2)	9.3 (2.7)	9.1 (2.5)	0.38 (0.28)	0.12 (0.76)	1.000	1.000
Hip rotation ROM (°)	11.7 (2.3)	11.4 (2.3)	15.2 (4.1)	16.2 (3.5)	3.5 (4.9)	4.8 (3.9)	−0.24 (0.51)	0.43 (0.22)	0.790	0.998
Hip rotation at IC (°)	−4.8 (3.5)	−5.5 (5.4)	−1.3 (4.7)	−6.9 (5.0)	3.5 (3.7)	1.4 (3.5)	0.50 (0.14)	0.60 (0.07)	0.645	0.119
Mean hip rotation—stand phase (°)	0.7 (3.0)	−3.2 (4.2)	1.9 (3.0)	−3.0 (4.4)	1.2 (1.7)	0.1 (1.6)	**0.85 ** **(0.004) ***	**0.90 ** **(<0.001) ***	**0.540**	**0.060**
Knee flexion ROM (°)	60.3 (4.4)	61.2 (4.2)	65.2 (4.2)	65.5 (3.7)	4.9 (4.4)	4.4 (3.4)	0.71 (0.03) *	0.43 (0.22)	0.892	0.860
Knee flexion at IC (°)	11.2 (5.1)	11.3 (5.7)	6.1 (4.3)	6.3 (4.9)	5.1 (2.7)	4.9 (3.6)	**0.81 ** **(0.008) ***	**0.81 ** **(0.008) ***	**0.855**	**0.750**
Maximal knee extension—stand phase (°)	3.8 (3.6)	2.5 (2.4)	4.5 (3.8)	4.2 (4.1)	0.7 (3.1)	1.7 (3.0)	0.53 (0.12)	0.76 (0.02) *****	0.086	0.413
Maximal knee flexion (°)	63.4 (2.0)	63.4 (3.9)	68.0 (4.2)	67.8 (4.3)	4.7 (3.3)	4.5 (2.4)	0.53 (0.12)	0.66 (0.04) *	0.943	0.852
Ankle flexion ROM (°)	29.3 (3.0)	28.9 (5.2)	30.9 (3.4)	33.1 (4.4)	1.6 (3.9)	4.2 (7.7)	0.22 (0.537)	−0.37 (0.296)	0.290	0.684
Ankle flexion at IC (°)	−1.5 (3.7)	−3.0 (2.5)	−9.0 (2.6)	−6.3 (2.5)	7.5 (1.7)	3.3 (1.8)	0.92 (<0.001) *	**0.81 ** **(0.008) ***	**0.999**	**0.959**
Maximum stance dorsiflexion (°)	17.0 (2.3)	17.0 (3.7)	12.2 (2.4)	13.5 (2.0)	4.8 (1.9)	3.5 (2.8)	0.55 (0.10)	**0.7** ** (0.03)***	0.999	0.952
Maximum swing dorsiflexion (°)	8.1 (2.8)	7.7 (3.3)	−3.1 (1.6)	0.5 (2.4)	11.1 (3.6)	7.1 (3.6)	−0.43 (0.22)	0.1 (0.79)	1.000	0.999
Maximum plantar flexion (°)	−12.3 (4.0)	−11.9 (6.3)	−18.7 (3.4)	−19.6 (4.8)	6.4 (4.1)	7.70 (7.45)	0.19 (0.608)	−0.02 (0.973)	0.998	0.966
Foot progression angle (°)	9.4 (3.7)	10.6 (5.3)	9.9 (4.8)	7.5 (5.8)	0.4 (4.0)	3.1 (3.2)	0.59 (0.08)	0.75 (0.02) *	0.062	0.350
Foot lift-off angle (°)	60.7 (5.2)	58.4 (4.2)	58.6 (6.8)	57.8 (7.8)	2.2 (5.8)	0.6 (8.0)	0.62 (0.06)	0.13 (0.73)	0.162	0.057
Foot landing angle (°)	13.7 (2.5)	14.4 (2.6)	8.6 (3.3)	8.4 (4.6)	5.2 (2.3)	6.0 (4.8)	0.65 (0.05) *	0.02 (0.97)	0.998	0.987

ROM—range of motion, IC—initial contact, * *p*-value < 0.05, bold letters indicate *p*-value < 0.05 on both sides.

**Table A2:** Comparison of the kinematic parameters between the WTC and WAC in the marker-based system.

Parameter	WTC		WAC		Difference		Spearman Correlation		Power	
	Right	Left	Right	Left	Right	Left	Right	Left	Right	Left
	Mean (SD)	Mean (SD)	Mean (SD)	Mean (SD)	Mean (SD)	Mean (SD)	Corr (*p*-value)	Corr (*p*-value)		
Stride length (m)	1.31 (0.12)	1.31 (0.13)	1.30 (0.12)	1.30 (0.12)	0.01 (0.04)	0.01 (0.04)	**0.94 ** **(<0.001) ***	**0.94 ** **(<0.001) ***	**0.056**	**0.056**
Step width (m)	0.09 (0.02)	0.10 (0.02)	0.09 (0.02)	0.09 (0.02)	0.00 (0.01)	0.01 (0.02)	**0.78 ** **(0.01) ***	**0.65 ** **(0.05) ***	**0.050**	**0.050**
Step length (m)	0.65 (0.06)	0.67 (0.07)	0.65 (0.06)	0.65 (0.06)	0.01 (0.02)	0.01 (0.02)	**0.95 ** **(<0.001) ***	**0.93 ** **(<0.001) ***	**0.050**	**0.157**
Gait speed (m/s)	1.24 (0.15)	1.25 (0.15)	1.24 (0.14)	1.23 (0.13)	0.00 (0.03)	0.01 (0.05)	**0.99 ** **(<0.001) ***	**0.92 ** **(<0.001) ***	**0.050**	**0.069**
Mean pelvis tilt (°)	−1.1 (7.2)	−1.0 (7.0)	−2.0 (7.4)	−2.1 (7.3)	0.9 (0.8)	1.1 (0.9)	**0.96 ** **(<0.001) ***	**0.96 ** **(<0.001) ***	**0.064**	**0.072**
Mean pelvis obliquity (°)	−1.5 (2.0)	1.4 (2.0)	−1.5 (2.0)	1.5 (2.0)	0.0 (0.1)	0.0 (0.2)	**1.00 ** **(<0.001) ***	**0.99 ** **(<0.001) ***	**0.050**	**0.052**
Pelvis obliquity ROM (°)	8.3 (2.8)	8.3 (2.9)	8.3 (2.6)	8.3 (2.6)	0.0 (0.6)	0.0 (0.7)	**0.95 ** **(<0.001) ***	**0.94 ** **(<0.001) ***	**0.050**	**0.050**
Pelvis obliquity at IC (°)	−3.1 (3.0)	−0.2 (2.7)	−3.2 (3.0)	−0.4 (2.6)	0.2 (0.2)	0.2 (0.3)	**0.99 ** **(<0.001) ***	**0.99 ** **(<0.001) ***	**0.051**	**0.055**
Pelvis rotation ROM (°)	11.3 (3.9)	11.4 (3.7)	11.0 (3.8)	10.8 (4.0)	0.2 (1.6)	0.6 (1.5)	**0.87 ** **(0.003) ***	**0.87 ** **0.003) ***	**0.056**	**0.0726**
Mean pelvis rotation (°)	−0.5 (1.8)	0.6 (2.0)	−0.7 (1.9)	0.6 (1.8)	0.1 (0.7)	0.0 (0.7)	**0.77** **(0.01) ***	**0.78** **(0.01) ***	**0.124**	**0.050**
Hip flexion ROM (°)	43.4 (4.6)	41.2 (8.8)	43.3 (5.0)	41.4 (8.9)	0.1 (1.7)	0.1 (1.5)	**0.93 ** **(<0.001) ***	**0.94 ** **(<0.001) ***	**0.050**	**0.050**
Hip flexion at IC (°)	26.5 (9.2)	26.4 (8.0)	25.5 (8.6)	24.9 (8.5)	1.0 (1.1)	1.5 (1.3)	**0.98 ** **(<0.001) ***	**0.99 ** **(<0.001) ***	**0.062**	**0.081**
Maximal hip extension (°)	−15.0 (8.8)	−13.9 (10.6)	−16.1 (9.0)	−15.4 (10.3)	1.0 (1.6)	1.5 (1.3)	**0.95 ** **(<0.001) ***	**0.95 ** **(<0.001) ***	**0.064**	**0.069**
Hip abduction ROM (°)	13.7 (3.0)	14.6 (4.0)	14.1 (3.1)	14.7 (3.9)	0.4 (0.8)	0.1 (1.0)	**0.94 ** **(<0.001) ***	**0.95 ** **(<0.001) ***	**0.066**	**0.050**
Hip abduction at IC (°)	−2.8 (2.0)	−0.6 (4.0)	−2.4 (2.6)	−1.0 (3.6)	0.5 (1.0)	0.4 (0.7)	**0.96 ** **(<0.001) ***	**0.98 ** **(<0.001) ***	**0.077**	**0.060**
Hip rotation ROM (°)	11.5 (2.8)	11.0 (3.4)	12.7 (2.2)	11.9 (3.4)	1.2 (1.3)	0.8 (0.7)	**0.85 ** **(0.004) ***	**0.92 ** **(<0.001) ***	**0.265**	**0.117**
Hip rotation at IC (°)	−4.6 (4.9)	−7.8 (5.8)	−4.4 (4.7)	−8.0 (5.9)	0.2 (0.8)	0.2 (1.8)	**0.99 ** **(<0.001) ***	**0.95 ** **(<0.001) ***	**0.052**	**0.051**
Mean hip rotation—stand phase (°)	−2.0 (4.0)	−4.4 (6.0)	−1.5 (3.8)	−4.5 (5.7)	0.5 (0.9)	0.1 (1.0)	**0.95 ** **(<0.001) ***	**0.93** ** (<0.001) ***	**0.205**	**0.052**
Knee flexion ROM (°)	58.5 (3.2)	58.5 (4.0)	58.3 (3.0)	58.9 (3.8)	0.2 (1.2)	0.4 (1.0)	**0.87 ** **(0.003) ***	**0.95 ** **(<0.001) ***	**0.053**	**0.059**
Knee flexion at IC (°)	14.2 (4.8)	14.3 (3.4)	12.9 (3.9)	12.6 (3.5)	1.3 (1.5)	1.7 (0.7)	**0.99 ** **(<0.001) ***	**0.94 ** **(<0.001) ***	**0.133**	**0.286**
Maximal knee extension—stand phase (°)	8.7 (2.9)	8.6 (3.9)	8.5 (2.7)	7.7 (3.6)	0.2 (0.7)	0.9 (1.0)	**0.92 ** **(<0.001) ***	**0.92 ** **(<0.001) ***	**0.079**	**0.384**
Maximal knee flexion (°)	65.2 (2.6)	65.3 (3.4)	64.5 (2.6)	64.8 (3.2)	0.6 (0.9)	0.5 (1.2)	**0.93 ** **(<0.001) ***	**0.90 ** **(<0.001) ***	**0.119**	**0.071**
Ankle flexion ROM (°)	28.7 (4.0)	25.9 (4.4)	28.7 (4.0)	25.5 (4.1)	0.0 (1.1)	0.3 (1.3)	**0.96 ** **(<0.001) ***	**0.96 ** **(<0.001) ***	**0.050**	**0.058**
Ankle flexion at IC (°)	1.9 (2.6)	4.4 (1.8)	2.4 (2.6)	4.9 (1.9)	0.5 (0.5)	0.5 (0.4)	**0.90 ** **(<0.001) ***	**0.99 ** **(<0.001) ***	**0.085**	**0.063**
Maximum stance dorsiflexion (°)	16.9 (3.4)	18.4 (3.5)	16.6 (3.3)	18.1 (3.7)	0.3 (0.4)	0.3 (0.9)	**0.96** ** (<0.001) ***	**0.94 ** **(<0.001) ***	**0.145**	**0.103**
Maximum swing dorsiflexion (°)	9.4 (1.7)	11.3 (2.7)	9.5 (1.7)	11.7 (2.4)	0.1 (0.6)	0.3 (0.9)	**0.98 ** **(<0.001) ***	**0.94 ** **(<0.001) ***	**0.133**	**0.229**
Maximum plantar flexion (°)	−11.8 (5.1)	−7.4 (6.0)	−12.0 (5.1)	−7.4 (5.5)	0.2 (1.3)	0.0 (2.0)	**1.00 ** **(<0.001) ***	**0.93 ** **(<0.001) ***	**0.051**	**0.051**
Foot progression angle (°)	10.2 (3.1)	10.0 (5.3)	9.4 (3.7)	10.6 (5.3)	0.8 (1.8)	0.6 (1.4)	**0.88** ** (0.002) ***	**0.96 ** **(<0.001) ***	**0.101**	**0.062**
Foot lift-off angle (°)	61.2 (5.3)	60.7 (5.3)	60.7 (5.2)	58.4 (4.2)	0.5 (2.8)	2.3 (3.5)	**0.77 ** **(0.01) ***	**0.67 ** **(0.04) ***	**0.058**	**0.233**
Foot landing angle (°)	11.9 (3.8)	12.4 (2.3)	13.7 (2.5)	14.4 (2.6)	1.9 (2.5)	2.0 (1.7)	0.81 (0.01) *	0.54 (0.11)	0.163	0.29

ROM—range of motion, IC—initial contact, * *p*-value < 0.05, bold letters indicate *p*-value < 0.05 on both sides.

**Table A3:** Spatial-temporal and kinematic parameters for the WAC between markerless and marker-based systems.

Parameter	Marker-Based		Markerless		Difference		Spearman Correlation		Power	
	Right	Left	Right	Left	Right	Left	Right	Left	Right	Left
	Mean (SD)	Mean (SD)	Mean (SD)	Mean (SD)	Mean (SD)	Mean (SD)	Corr (*p*-value)	Corr (*p*-value)		
Stride length (m)	1.30 (0.12)	1.30 (0.12)	1.30 (0.12)	1.28 (0.12)	0.01 (0.02)	0.02 (0.04)	**0.98** **(<0.001) ***	**0.89** **(0.001) ***	**0.050**	**0.467**
Step width (m)	0.09 (0.02)	0.09 (0.02)	0.12 (0.02)	0.11 (0.02)	0.02 (0.01)	0.02 (0.01)	**0.83** **(0.006) ***	**0.85** **(0.004) ***	**0.987**	**0.987**
Step length (m)	0.65 (0.06)	0.65 (0.06)	0.64 (0.06)	0.63 (0.07)	0.00 (0.01)	0.02 (0.05)	**0.96** **(<0.001) ***	**0.68** **(0.04) ***	**0.064**	**0.062**
Gait speed (m/s)	1.24 (0.14)	1.23 (0.13)	1.23 (0.14)	1.21 (0.13)	0.01 (0.02)	0.02 (0.04)	**0.99** **(<0.001) ***	**0.92 ** **(<0.001) ***	**0.053**	**0.053**
Mean pelvis tilt (°)	−2.0 (7.4)	−2.1 (7.3)	−0.8 (3.4)	−0.9 (3.3)	1.1 (7.8)	1.2 (7.7)	0.24 (0.51)	0.14 (0.71)	0.083	0.084
Mean pelvis obliquity (°)	−1.5 (2.0)	1.5 (2.0)	−0.0 (0.9)	0.3 (0.6)	1.5 (1.8)	1.2 (2.0)	0.52 (0.13)	0.37 (0.30)	0.683	0.479
Pelvis obliquity ROM (°)	8.3 (2.6)	8.3 (2.6)	17.4 (4.1)	18.3 (3.6)	10.0 (3.5)	10.0 (3.5)	0.60 (0.07)	0.47 (0.18)	0.999	1.000
Pelvis obliquity at IC (°)	−3.2 (3.0)	−0.4 (2.6)	−7.4 (2.7)	−7.6 (2.2)	4.2 (3.2)	7.2 (2.1)	0.19 (0.60)	0.64 (0.05)	0.984	0.250
Pelvis rotation ROM (°)	11.0 (3.8)	10.8 (4.0)	14.4 (3.5)	17.4 (4.6)	3.3 (6.2)	6.6 (5.5)	−0.47 (0.18)	0.21 (0.56)	0.744	0.989
Mean pelvis Rotation (°)	−0.7 (1.9)	0.6 (1.8)	−1.5 (2.1)	0.9 (1.8)	0.8 (2.8)	0.3 (2.2)	0.07 (0.87)	0.26 (0.47)	0.204	0.076
Hip flexion ROM (°)	43.3 (5.0)	41.4 (8.9)	43.9 (3.4)	41.5 (5.7)	0.6 (4.8)	0.1 (6.7)	0.26 (0.47)	0.66 (0.04) *	0.067	0.050
Hip flexion at IC (°)	25.5 (8.6)	24.9 (8.5)	29.9 (3.6)	27.2 (3.3)	4.4 (7.9)	2.3 (7.6)	0.42 (0.23)	0.58 (0.09)	0.383	0.142
Maximal hip extension (°)	−16.1 (9.0)	−15.4 (10.3)	−13.0 (3.0)	−13.0 (3.9)	3.0 (8.3)	2.4 (9.4)	0.28 (0.43)	0.45 (0.19)	0.198	0.118
Hip Abduction ROM (°)	14.1 (3.1)	14.7 (3.9)	21.2 (5.5)	23.7 (4.2)	7.2 (3.6)	9.0 (4.2)	0.78 (0.01) *	0.56 (0.10)	0.986	0.999
Hip Abduction at IC (°)	−2.4 (2.6)	−1.0 (3.6)	−7.0 (2.8)	−7.9 (2.2)	4.7 (3.1)	6.9 (3.0)	0.28 (0.43)	0.50 (0.14)	0.997	0.999
Hip rotation ROM (°)	12.7 (2.2)	11.9 (3.4)	15.2 (4.1)	16.2 (3.5)	2.5 (5.3)	4.3 (4.9)	−0.30 (0.41)	0.10 (0.79)	0.507	0.938
Hip rotation at IC (°)	−4.4 (4.7)	−8.0 (5.9)	−1.3 (4.7)	−6.9 (5.0)	3.1 (3.4)	1.1 (5.2)	0.66 (0.04) *	0.48 (0.17)	0.438	0.088
Mean hip rotation—stand phase (°)	−1.5 (3.8)	−4.5 (5.7)	1.9 (3.0)	−3.0 (4.4)	3.4 (2.7)	1.5 (5.3)	0.52 (0.13)	0.36 (0.31)	0.802	0.113
Knee flexion ROM (°)	58.3 (3.0)	58.9 (3.8)	65.2 (4.2)	65.5 (3.7)	6.9 (4.6)	6.6 (5.0)	0.03 (0.94)	0.30 (0.41)	0.999	0.998
Knee flexion at IC (°)	12.9 (3.9)	12.6 (3.5)	6.1 (4.3)	6.3 (4.9)	6.8 (3.3)	6.3 (3.1)	0.78 (0.01) *	0.61 (0.07)	0.996	0.981
Maximal knee extension—stand phase (°)	8.5 (2.7)	7.7 (3.6)	4.5 (3.8)	4.2 (4.0)	4.0 (2.8)	3.5 (3.1)	0.75 (0.02) *	0.25 (0.49)	0.993	0.562
Maximal knee flexion (°)	64.5 (2.6)	64.8 (3.2)	68.0 (4.2)	67.8 (4.3)	3.5 (4.7)	3.0 (5.3)	0.04 (0.92)	−0.02 (0.97)	0.765	0.590
Ankle flexion ROM (°)	28.7 (4.0)	25.5 (4.1)	30.9 (3.4)	33.1 (4.4)	2.3 (3.6)	7.6 (5.8)	0.53 (0.12)	0.02 (0.97)	0.384	0.999
Ankle flexion at IC (°)	2.4 (2.6)	4.9 (1.9)	−9.0 (2.6)	−6.3 (2.5)	11.4 (2.3)	11.2 (3.7)	0.43 (0.22)	−0.35 (0.33)	1.000	1.000
Maximum stance dorsiflexion (°)	16.6 (3.4)	18.1 (3.7)	12.2 (2.4)	13.5 (2.0)	4.4 (3.4)	4.6 (3.2)	0.33 (0.35)	0.54 (0.11)	0.946	0.985
Maximum swing dorsiflexion (°)	9.5 (1.7)	11.7 (2.4)	−3.1 (1.6)	0.5 (2.4)	12.6 (2.7)	11.2 (3.8)	−0.44 (0.20)	0.54 (0.11)	1.000	1.000
Maximum plantar flexion (°)	−12.0 (5.1)	−7.4 (5.5)	−18.7 (3.4)	−19.6 (4.8)	6.7 (5.2)	12.2 (6.7)	0.04 (0.92)	−0.13 (0.73)	0.986	0.999
Foot progression angle (°)	9.4 (3.7)	10.6 (5.3)	9.9 (4.8)	7.5 (5.8)	0.4 (4.0)	3.1 (3.2)	0.59 (0.08)	0.75 (0.02) *	0.062	0.350
Foot lift-off angle (°)	60.7 (5.2)	58.4 (4.2)	58.6 (6.8)	57.8 (7.8)	2.2 (5.8)	0.6 (8.0)	0.62 (0.06)	0.13 (0.73)	0.162	0.057
Foot landing angle (°)	13.7 (2.5)	14.4 (2.6)	8.6 (3.3)	8.4 (4.6)	5.2 (2.3)	6.0 (4.8)	0.65 (0.05) *	0.02 (0.97)	0.998	0.987

ROM—range of motion, IC—initial contact, * *p*-value < 0.05, bold letters indicate *p*-value < 0.05 on both sides.
